# Clustering individuals’ temporal patterns of affective states, hunger, and food craving by latent class vector-autoregression

**DOI:** 10.1186/s12966-022-01293-1

**Published:** 2022-05-21

**Authors:** Björn Pannicke, Jens Blechert, Julia Reichenberger, Tim Kaiser

**Affiliations:** 1 Department of Psychology, Centre for Cognitive Neuroscience, Paris-Lodron-University of Salzburg, Salzburg, Austria; 2 Department of Psychology, Clinical Psychology and Psychotherapy, University of Greifswald, Greifswald, Germany

**Keywords:** LCVAR, Latent class vector-autoregression, Cluster, Eating behaviour, Food craving, Hunger, Affect, Stress coping, Temporal relationships, Individual differences

## Abstract

**Background:**

Eating plays an important role in mental and physical health and is influenced by affective (e.g., emotions, stress) and appetitive (i.e., food craving, hunger) states, among others. Yet, substantial temporal variability and marked individual differences in these relationships have been reported. Exploratory data analytical approaches that account for variability between and within individuals might benefit respective theory development and subsequent confirmatory studies.

**Methods:**

Across 2 weeks, 115 individuals (83% female) reported on momentary affective states, hunger, and food craving six times a day. Based on these ecological momentary assessment (EMA) data we investigated whether latent class vector-autoregression (LCVAR) can identify different clusters of participants based on similarities in their temporal associations between these states.

**Results:**

LCVAR allocated participants into three distinct clusters. Within clusters, we found both positive and negative associations between affective states and hunger/food craving, which further varied temporally across lags. Associations between hunger/food craving and *subsequent* affective states were more pronounced than vice versa. Clusters differed on eating-related traits such as stress-eating and food craving as well as on EMA completion rates.

**Discussion:**

LCVAR provides novel opportunities to analyse time-series data in affective science and eating behaviour research and uncovers that traditional models of affect-eating relationships might be overly simplistic. Temporal associations differ between subgroups of individuals with specific links to eating-related traits. Moreover, even within subgroups, differences in associations across time and specific affective states can be observed. To account for this high degree of variability, future research and theories should consider individual differences in direction and time lag of associations between affective states and eating behaviour, daytime and specific affective states. In addition to that, methodological implications for EMA research are discussed.

**Supplementary Information:**

The online version contains supplementary material available at 10.1186/s12966-022-01293-1.

## Background

Over the last decades, eating behaviours have become an intensely researched topic, as they are associated with multiple physical health outcomes − e.g., cardiovascular diseases [1] and diabetes [2] − as well as mental health outcomes − e.g., eating disorders [3] and depression [4]. Thus, eating behaviours impact not only individuals’ quality of life but also health care systems [5, 6]. Although eating behaviours are strongly affected by homeostatic processes such as hunger or energy deficits, they can also be expressions of habits, lifestyle [7], and self-regulation [8]. Compared to traditional models that focus on independent homeostatic and hedonic processes, recent models (e.g., regarding appetite control) include ‘cross-talk’ of homeostatic, cognitive and reward mechanisms [9]. Therefore, eating can be viewed as a result of interactions between physiological, affective, and psychological variables. Central non-homeostatic variables influencing eating behaviours include food cravings [10], positive and negative emotions [11] as well as perceived stress [12] / stress coping [13, 14], among others. Given both the importance and the complexity of eating behaviours, several approaches within psychological research were developed to assess the nature and direction of associations between eating behaviour and *affective states* (hereinafter used as an umbrella term for emotions as well as experiencing and coping with stress [15]). In the following, we will briefly review these approaches and highlight perspectives on variability in associations between eating behaviour and affective states.

### From the main effect model to the individual difference model of affect-eating relationships

For several decades, effects of stress on food intake have been documented in both rodent and human studies: While some studies observed an increase in food intake after a stressor, others described the opposite, i.e., a decrease in food intake in response to a stressor [16]. Such effects have been explained in terms of ‘*main effect models’*, meaning that, depending on the theory, stress either increases or decreases food intake. To illustrate, different models have tried to explain these effects psychologically (e.g., eating more to alleviate negative feelings [17]) and physiologically (e.g., acute stress leading to a fight-or-flight based reduction of appetite, thus, decreased eating vs. chronic stress leading to increased eating driven by the hypothalamic-pituitary-adrenal axis [16]). Yet, human individuals can differ notably in the direction and strength of such stress-eating relationships, which led to the *individual difference model* [18]: Some individuals increase their eating behaviour in reaction to stress or negative affect, while others decrease it or show no effect on eating, resulting in pronounced between-subject variability that can be (partially) explained by individual characteristics. To assess this variability, trait-level questionnaires were developed to capture individual differences, such as the Dutch Eating Behavior Questionnaire (DEBQ [19]) tapping into a range of negative emotional states and measuring to what degree these states are reported to increase subsequent craving or eating.

However, as different affective states are related to different physiological responses, their effects on eating behaviour likely differ as well. Thus, further differentiation on the level of affective states became necessary: First, stress has been differentiated from various negative emotions (e.g., anxiety or anger) in recently developed psychometric scales: The Salzburg Stress Eating Scale (SSES [20]) and the Salzburg Emotional Eating Scale (SEES [21]) revealed that stress eating appears to be a distinct concept compared to emotional eating. Second, within emotions, negative and positive emotions need to be distinguished from each other and even within negative emotions, highly arousing emotions such as anger or anxiety showed distinct associations with eating in comparison to low arousal emotions such as sadness [21]. Thus, as the field of stress-eating and emotional eating matured, the number of possible affect-eating relationships increased and with that theories and approaches became more complex to account for this variability [17, 22].

### Individual and temporal differences: ecological momentary assessment studies

While trait questionnaires revealed strong between-subject variability and marked differentiation on the level of affective states, they do not account for momentary and contextual influences and circadian patterns that characterise naturalistic eating behaviours. Instead of relying on one-time, trait questionnaires inquiring about ‘general’ or ‘typical’ affect-eating patterns [17, 23], ecological momentary assessment (EMA) can survey affective states and eating behaviour multiple times a day in natural settings. Typically smartphone-based, EMA gathers ‘real-time’ and ‘real-world’ data through repeated short questions across the day, thereby limiting retrospective memory biases [24]. Importantly, multiple assessments per day allow a *lagging* of variables: relating one variable at measurement point t_x_ (e.g., negative mood at noon) to another variable at a subsequent measurement point t_x + 1_ (e.g., food craving at 4 pm) thus uncovering temporal and potentially causal effects of one variable on another. Studies using EMA in addition to questionnaire data have for instance shown that relationships between stress and eating behaviour can differ based on the time point and aggregation level (i.e., associations lagged within a day vs. overall day level associations after aggregation [25]).

### Accounting for variability within and between individuals in EMA data: multilevel models and network models

Several analytical approaches to the variability inherent in EMA data have been taken. Theory-driven analytical approaches using multilevel modelling (MLM [26]) relate momentary (within-subject, *Level 1*) variables to each other, while the variables and the direction of influence/lagging (e.g., affect influences eating instead of eating influencing affect) are predetermined by the researcher and thus also lead to a limited perspective on associations assumed. This statistical technique also allows for the simultaneous modelling of trait level questionnaires (between-subject, *Level 2*), e.g., stress and eating behaviour are associated within participants with high scores on the SSES [25]. Exploratory approaches, by contrast, do not constrain the choice of variables that vary within individuals: Vector-autoregressive (VAR) models allow calculating associations of variables among each other based on their previous values and by considering the respective autoregression of X_t_ on X_t + 1_ (each variable predicts itself on a future time point) [27]. Besides contemporaneous and between-subject analyses, multilevel vector-autoregression (mlVAR [28]) can fit a *temporal network* model based on EMA (time-series) data. Thus, directed (i.e., lagged) and unique (i.e., partial correlative) effects of X_t_ on Y_t + 1_ and Y_t_ on Z_t + 1_ (etc) can be obtained. The temporal network structure is estimated by considering all variables of interest and their unique lagged associations among each other [27]. A network analysis study by our group found that higher anticipated stress coping preceded higher goal-congruent eating [14]. Novel findings revealed through network models illustrate how exploratory analytical approaches can influence theory building.

### Exploring similar patterns: latent class vector-autoregressive modelling

Although such a temporal network structure is rather comprehensive, it lacks the consideration of between-subject differences in the identified within-subject temporal relationships. MLM, by contrast, requires a theory-driven choice for Level 2 (between-subject) data as potential moderators of associations (e.g., trait questionnaires). Until recently, it was not possible to determine between-subject variability in within-subject associations in a data driven manner. The development of latent class vector-autoregression (*LCVAR*) closes this gap by exploring latent classes of individuals (i.e., distinct subgroups within a sample) based on similarities in lagged *Level 1* associations, thus based on similar temporal dynamics [29]. Furthermore, LCVAR provides opportunities to explore *within*-subject variability: Several lags can be considered, for instance, stress at t_x_ might be associated with other variables not only at t_x + 1_ but also at t_x + 2_ or even at t_x + 3_, while the strength and direction of these relationships might differ between lags. This important complexity has already been noted in EMA research [25] but has not been addressed comprehensively so far. In the following, we will briefly describe LCVAR, its advantages and its potential for affective eating behaviour research.

### Latent class vector-autoregressive models

LCVAR models can be characterised as multilevel models based on repeated measures (i.e., occasions) at *Level 1* that are nested within individuals at *Level 2*. In LCVAR, time-series Level 1 data provide covariate coefficients (concerning trends and exogenous variables, such as *time*) and a lagged VAR model (concerning dynamics). Using VAR coefficients, dynamic processes can be investigated as effects of the endogenous variables at t_x_ on themselves at t_x + 1_, resulting in autoregressive and cross-regressive coefficients. Time-series data are handled by maximum likelihood estimation which allows modelling intensive and unequally long time-series data. LCVAR utilises the Expectation Maximization (EM) algorithm [30] to estimate probabilistic latent class membership and class-wise time-series parameters. Thus, at Level 2, LCVAR models latent classes (i.e., clusters) with fixed coefficients for each cluster. The method utilises an adaptive model estimation which can result in different numbers of lags for each cluster. Setting an information criterion such as the Hannan-Quinn (HQC [31]) or the Akaike information criterion (AIC) enables a model selection of different cluster solutions (i.e., solutions with different numbers of clusters and different numbers of lags). Lastly, by considering interpretability and estimation convergence, a final cluster solution is chosen. Such a solution contains several distinct clusters with (potentially different) lag orders. Each individual is assigned to one cluster only. For detailed information on LCVAR, please also see the original paper [29].

### Aims of the present study

The goal of our study was to apply the recently developed exploratory method of latent class vector-autoregressive modelling [29] to naturalistic affective and eating behaviour research. We aimed to explore between-subject differences in the dynamics of affective states, hunger, and food craving (i.e., urges to eat specific foods [32]). Such temporal associations might be similar across some individuals and could thus form *clusters* of dynamic patterns. Given the exploratory approach, hypotheses are tentative only. Overall, we expect high variability regarding a) specific associations (coefficients) between specific affective and eating-related variables (e.g., anger might relate differently to subsequent craving than sadness does, or craving might increase subsequent anger), b) regarding temporal resolution of relationships (based on different lags), and c) regarding differences between clusters (positive associations in one cluster, negative associations in another) as suggested by the respective questionnaire-based research reviewed above. Lastly, we aimed to characterise clusters using visualisations of daytime courses of all variables, cross-sectional questionnaire data and EMA completion rates.

## Methods

### Procedure

The data analysed in the present study were taken from a mid-study subsample of individuals who took part in a randomised controlled trial (RCT) that was preregistered at the German register of clinical studies (DRKS, DRKS00017493). The RCT was based on a three-week EMA study during which the active group received eating behaviour related short messages. Data of both groups (i.e., active and control group) were included in the present analyses. For the exploratory LCVAR, data are based on 14 days of EMA that constituted the ‘*main phase’* (which served as the ‘treatment’-phase for the active group, respectively as the control phase) of the superordinate study. Individuals were recruited for the RCT through study announcements via e-mail at several universities in Austria and Germany, Facebook groups, Twitter, and by word of mouth. Similar to previous study inclusion criteria [14], agreeing to one of the following two questions enabled individuals to take part in the study: 1) ‘*Do you currently pay attention to your nutrition to maintain or reduce your body weight’?* and 2) *‘Do you currently cut down on your food intake to maintain or reduce your body weight’?* Thus, generally, diet-interested individuals were recruited. Furthermore, it was required to own an Android smartphone to install the app for the EMA. In the beginning, participants reported demographic variables and answered several trait questionnaires (see *Trait questionnaires**).* After that, participants were instructed on how to use our customised smartphone app ‘*SmartEater*’ that was installed manually on participants’ Android smartphones and that served as the assessment tool for the EMA. At the beginning of the study, participants received written and oral information on the study and signed an informed consent form according to the ethics committee at the University of Salzburg, Austria that granted ethical approval (EK-GZ: 37/2018). After the EMA period, participants answered questions regarding subjective reactivity: 1) “How much did the assessment itself change your eating behaviour?” (0 = negatively, 50 = unchanged, 100 = positively) and 2) “Did you eat differently than you would normally have in some situations because of the app answers?” (0 = not at all, 50 = partly, 100 = very much). Both questions were answered on a scale from 0 to 100 in steps of ten. At the end of the study, participants received visualised feedback of their EMA data and financial compensation or course credits for their study participation. Please note that other parts of the dataset have previously been analysed and published [33].

### EMA measures

EMA data used in this study were gathered by signal-contingent sampling: Six times a day (9:00 a.m., 11:30 a.m., 2:00 p.m., 4:30 p.m., 7:00 p.m., 9:30 p.m.) participants received app notifications (‘beeps’) on their smartphones. Each time, they were asked to rate their momentary affects, stress (coping), hunger and food craving on a horizontal, continuous rating slider from 0 (= not at all) to 100 (= very much). For a list of variables measured but not used in the present study, please see the ‘List of additional variables’ in the supplementary materials. Items used in this study have also been used in previous studies [14]: The affect items were partially taken from the *Positive and Negative Affect Schedule* (*PANAS*) with additional items added that feature different thresholds or levels of arousal. Positive affect items included ‘cheerful’, ‘enthusiastic’, ‘relaxed’, ‘calm’ and ‘active’, whereas negative affect items included ‘irritated’, ‘worried’, ‘depressed’, ‘bored’ and ‘nervous / stressed’. Stress coping was also assessed similar to previous research [14]: Participants reported their present stress coping efficacy (‘*Do you feel that you are on top of things?*’) and their anticipated stress coping efficacy (‘*Do you feel that you can cope with all upcoming things that you will have to do?*’). The questions were worded in the style of the Perceived Stress Scale (PSS [34]) and were adapted to a state level. Furthermore, hunger and food cravings were also assessed as in previous studies [35]: ‘*How strong is your hunger right now?*’ (hunger) and ‘*How strong is your desire to eat certain foods right now?*’ (food cravings). Participants were instructed that ‘certain foods’ refer to specifically tasty foods. For reasons of safety (e.g., while driving) and practicability, participants had up to 1 h to answer the questions of each EMA beep. Thereafter, the respective EMA questionnaire disappeared and was registered as missing values.

### Trait questionnaires

Before the EMA period, participants answered several online questionnaires that were presented via the platform LimeSurvey [36]. The following trait-questionnaires were used for the present study and aimed to capture between-subject variability: the three subscales (emotional eating, restrained eating and external eating) of the Dutch Eating Behavior Questionnaire (DEBQ [19]) that queries different influences on eating, the Food Cravings Questionnaire Trait reduced (FCQ-T-r [37]) which assesses trait-level experiences of food craving, the Salzburg Stress Eating Scale (SSES [20]), and the four subscales (anger, anxiety, happiness, sadness) of the Salzburg Emotional Eating Scale (SEES [21]). The SSES and SEES assess how individuals perceive stress, respectively specific emotions to influence the amount of food they consume.

### Latent class vector-autoregressive modelling

All analyses were run in *R* (version 4.0.3) using *RStudio* (version 1.3.1093). We analysed inter-individual patterns of associations among the fourteen variables described above (i.e., five positive affect items, five negative affect items, two stress coping items, one hunger item and one food craving item). For the analyses, we included participants with high adherence/compliance, i.e., completing at least 60% of all EMA questionnaires (i.e., at least 50 out of 84 EMA questionnaires across the 14 days). Having at least 50 observations per person is recommended for running an LCVAR [29]. Missing values were imputed using ‘Multivariate Imputation by Chained Equations’ (MICE), as implemented in the *mice* package [38], with 500 iterations of a single imputation. All functions necessary for the LCVAR were derived from *GitHub* [39]. Since the present study is the first to apply LCVAR to eating behaviour research, our theoretical assumption of cluster numbers is based on the literature reviewed above, which allows, among others, two hypothetical (general) distinctions: Firstly, individuals might be grouped together based on a ‘dichotomous’ main effect model (i.e., observable associations vs. no observable associations between affective states and eating behaviour). Secondly, individuals might also be clustered into three groups based on similar ‘individual differences’ (e.g., direction of coefficients) regarding these associations (i.e., no observable associations vs. mostly positive associations vs. mostly negative associations between affective states and eating behaviour). Previous studies have reported similar subgroup distinctions regarding stress and the amount of food consumed [20, 40]. Given these theoretical assumptions, the exploratory nature of our study, and the present (limited) sample size we thus set the minimum number of clusters to two and the maximum number of clusters to three. Regarding cluster memberships, we decided to require each cluster to have at least two members. Moreover, we set the lowest lag required to one and the highest potential lag to three which considers the assessment frequency given and provides a plausible timeframe for temporal associations of affective states and eating behaviour. As previously suggested [29], we used the extended Hannan-Quinn information criterion (HQC) for LCVAR as it enables finding a model solution that ideally combines lags and memberships of clusters. Furthermore, we oriented our LCVAR settings towards the ones of the original article describing the analysis [29]. We set the number of pseudo-random starts (i.e., randomly selecting *k* individuals as cluster centres) of the EM algorithm to 15 for fitting each potential model, while rational starts (i.e., *k*-means partitioning based on individuals’ VAR and coefficients of covariates) of the algorithm were also used. When posterior probabilities of cluster memberships were reset, every element of Sigma was increased by 10. After every start of the EM algorithm, 50 EM-iterations were allowed before this EM-start had to terminate. Lastly, to determine the convergence of an EM-start, the convergence criterion of the log-likelihood was set to 1e-07. In summary, our present LCVAR combined each lag order, i.e., one, two or three lag(s) for both numbers of clusters, i.e., two or three clusters. Each of these combinations was based on its own statistical model. The start of the EM algorithm was based on 15 pseudo-random initialisations, k-means based rational initialisations and previous solutions. After a maximum of 50 EM-iterations or convergence of the likelihood, each EM-start led to a solution, eventually resulting in multiple solutions out of which the ideal solution for two, respectively, three clusters (across all lag combinations and EM-starts) was chosen based on interpretability (i.e., theoretically plausible differentiation between patterns of lagged associations) and the HQC. Resting upon our sampling plan and frequency, we additionally used the time of day at which participants answered EMA questionnaires as the exogenous variable in the analyses. The exogenous variable allows plotting the mean values of each endogenous variable (i.e., each item) at all six time points (‘beeps’) for all clusters separately which allows insights into daytime courses of the items. For more details regarding potential parameters, settings and optional arguments of LCVAR, please see the original paper [29]. To support the understanding of the meaning of the present LCVAR results, we additionally added a simulated LCVAR to the supplementary materials (see 'Simulated random data LCVAR') for which we used the same settings as described above but randomised data, as suggested by a reviewer of this paper.

## Results

### Participants’ descriptive statistics

The initial sample of the present study consisted of 136 participants. Twenty-one participants were excluded from the analyses because they completed less than 60% of all EMA questionnaires. Thus, 115 individuals (i.e., a total number of 9660 Level 1 occasions) were included in the LCVAR. Because of missing descriptive data for one participant, descriptive statistics and additional analyses are based on 114 participants, of whom 95 were female (83.3%) and 19 were male (16.7%). The mean age was 23.2 years (*SD* = 3.81 years, range = 18–40 years). The group allocation of individuals was rather balanced with 56 participants out of the treatment group (49.12%) and 58 participants out of the control group (50.88%). The mean BMI was 23.32 kg/m^2^ (*SD* = 3.72 kg/m^2^, range = 17.8–37.65 kg/m^2^). Participants reported a low to moderate influence of the assessment itself (*M* = 35.4, *SD* = 23.4, *N* = 112) and of their app answers (*M* = 30.8, *SD* = 26.5, *N* = 112) on their eating behaviour.

### Latent class vector-autoregression

**Fig. 1:**
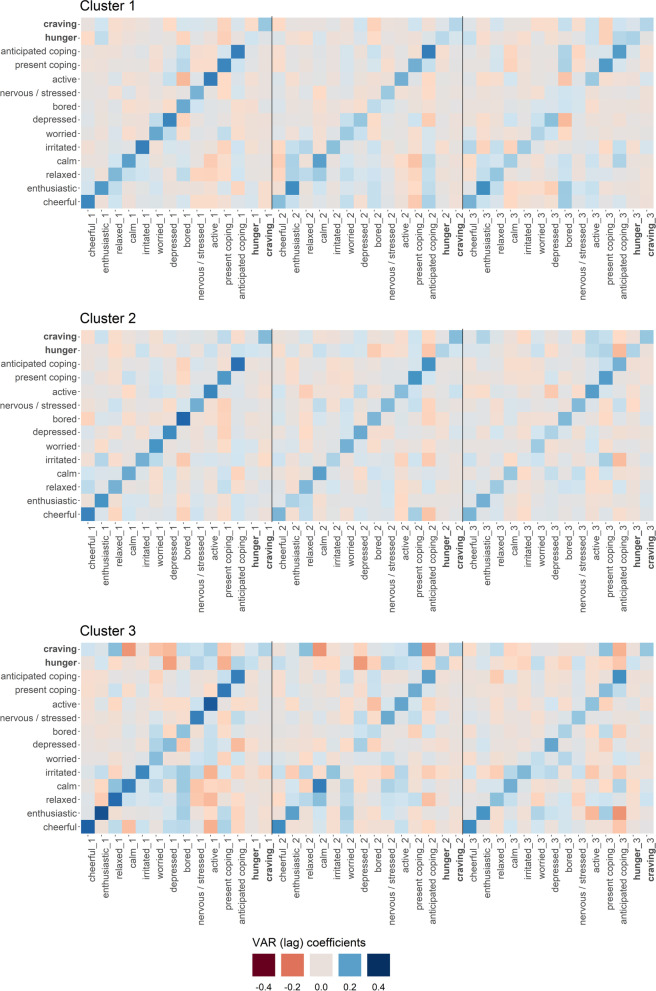
Heatmaps of the selected cluster solution. *Note.* VAR = vector-autoregressive, _x = x lags. Vertical lines were added to visualise the beginning of a new lag. Words are in bold for visualisation purposes only. For full item meanings please see ‘EMA measures’ in the Methods section

**Fig. 2:**
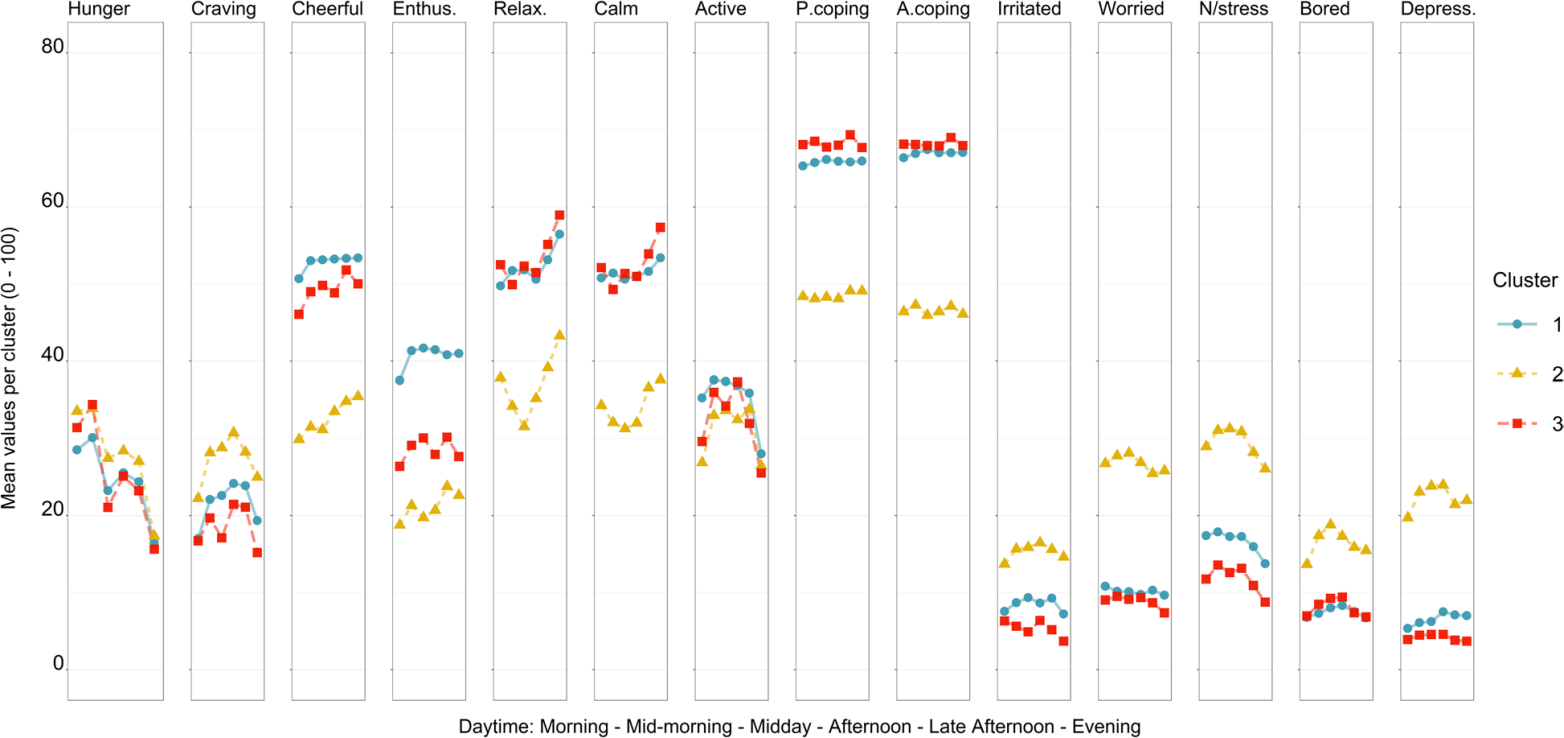
Mean values of all fourteen endogenous variables for all three clusters. *Note.* X-axis: daytime, y-axis = mean values of the respective clusters. Range of all item scales: 0 to 100. Enthus. = Enthusiastic, Relax. = Relaxed, P.coping = Present stress coping, A.coping = Anticipated stress coping, N/stress. = Nervous / stressed, Depress. = Depressed. Colours are based on colour palettes from the wesanderson package [41]

The LCVAR resulted in two cluster solutions. *Solution 1* contained two clusters with three lags each. *Solution 2* contained three clusters, also with three lags each. Given the slightly smaller HQC of *Solution 2* (HQC_2_ = 77.72) compared to *Solution 1* (HQC_1_ = 77.86), we decided to further investigate Solution 2 containing three clusters. Moreover, as it may be reasonable to obtain clusters that differ regarding the ‘roles’ of hunger and food craving (i.e., negative/positive, respectively, small/large coefficients) in the respective clusters, we chose the solution containing three clusters displayed as heatmaps in Fig. 1 for all further analyses. Additionally, this solution appeared to capture variability both within and between clusters. Eight EM-iterations were repeated before the forced end of the algorithm. Cluster membership proportions were distributed as follows: Cluster 1: 46.1%, Cluster 2: 31.3%, Cluster 3: 22.6%. Since we used the time of day at which participants answered EMA questionnaires as the exogenous variable in our LCVAR, we plotted the mean values of each endogenous variable (item) at all six daily time points for all clusters separately (see Fig. 2).

In the following, we will focus specifically on *variability* concerning hunger and craving-related associations (see the *first two rows* as well as the *two columns before the vertical ‘lag lines’* in the cluster heatmaps in Fig. 1). The LCVAR resulted in three subgroups in which the direction and strength of coefficients appear to differ both across lags within clusters and – expectedly - between clusters. The ‘daytime courses’ in Fig. 2 display *Cluster 2* as the most fluctuating of the clusters regarding the endogenous variables, with the lowest mean positive and highest mean negative affects in general. Moreover, *Cluster 2* also showed the strongest increase in cravings, with high values especially in the afternoon. By contrast, *Cluster 3* showed the strongest but also the most differentiated associations between craving and hunger on the one hand and subsequent affective states on the other hand: unexpectedly, the associations of affective variables with subsequent hunger and food craving were very small (‘emotional eating’, see last two columns in each lag quadrant of the heatmap). By contrast, the reverse direction of association emerged in *Cluster 3* (‘emotional about eating’, first two rows of the heatmap). Food craving and hunger were associated with subsequent affective states in different ways: Food craving showed negative coefficients for subsequently feeling calm (β_lag_1_ = −.154, β_lag_2_ = −.156) and anticipated stress coping (β_lag_2_ = −.157) while also positive associations with subsequent positive emotions were observed, e.g., cheerful (β_lag_1_ = .049) and active (β_lag_1_ = .100). Also in Cluster 3, hunger showed negative coefficients with subsequently feeling depressed (β_lag_1_ = −.140, β_lag_2_ = −.149): experiencing more hunger preceded feeling less depressed. For all associations between affective states, hunger, and food craving please also see Fig. 1, respectively Fig. 2 (for daytime courses) as well as the ‘Coefficients tables’ in the supplementary materials.

### Characterising clusters using questionnaires and completion rates

**Fig. 3:**
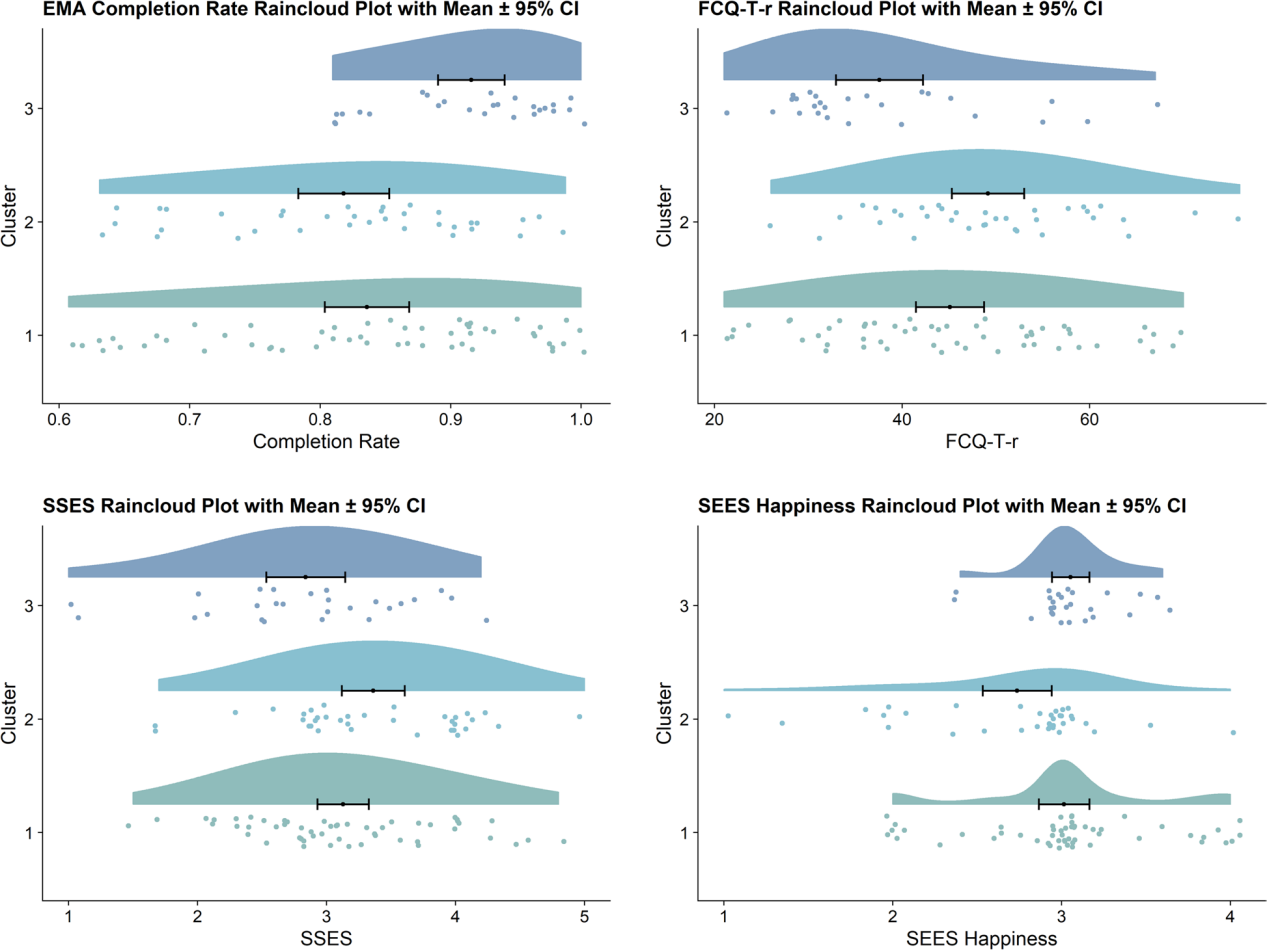
Raincloud plots of cross-sectional data of the three clusters. Note. *N* = 114. Only variables that revealed significant differences between clusters are displayed (see also Table 1 for all ANOVA results). Plots include cluster specific means, 95% confidence intervals, information on distributions as well as added random jitter. Y-axis = clusters, x-axis = respective score. CI = confidence interval, EMA = ecological momentary assessment, FCQ-T-r = Food Cravings Questionnaire Trait reduced, SSES = Salzburg Stress Eating Scale, SEES Happiness = Salzburg Emotional Eating Scale (Happiness Subscale). Colours are based on colour palettes from the nord package [42]. For more information on raincloud plots, please see the original paper [43]

After obtaining the algorithm-based cluster solutions, we aimed to characterise the clusters by analysing potential Level 2 (i.e., between-subject data) differences between clusters by means of univariate ANOVAs. Table 1 displays the results of the ANOVAs including the above-mentioned questionnaires (see *Trait questionnaires* in the *Methods* section) and completion rates. Post hoc contrasts revealed specific significant differences between clusters regarding cross-sectional data: Cluster 3 had the significantly lowest mean FCQ-T-r score (mean = 37.58) compared to Cluster 1 (Tukey multiple comparisons of means (Tukey HSD), ∆_(3-1)_ = − 7.52, *p* = .031) and Cluster 2 (Tukey HSD, ∆_(3-2)_ = − 11.57, *p* = .001). Moreover, Cluster 3 also had the significantly highest mean compliance (mean = 0.92) compared to Cluster 1 (Tukey HSD, ∆_(3-1)_ = 0.08, *p* = .004) and Cluster 2 (Tukey HSD, ∆_(3-2)_ = 0.10, *p* = .001). In addition, Cluster 3 had a significantly lower mean SSES score (mean = 2.84) compared to Cluster 2 (Tukey HSD, ∆_(3-2)_ = − 0.52, *p* = .022), however it did not differ significantly from Cluster 1 (Tukey HSD, ∆_(3-1)_ = − 0.29, *p* = .246). Lastly, Cluster 2 showed the lowest mean SEES happiness score (mean = 2.74) compared to Cluster 1 (Tukey HSD, ∆_(2-1)_ = − 0.28, *p* = .038) and Cluster 3 (Tukey HSD, ∆_(3-2)_ = 0.32, *p* = .049). Please also see Fig. 3 that displays *raincloud plots* illustrating the mean, confidence intervals, and information on distributions of the four significant differences in cross-sectional data (i.e., compliance, SSES, SEES happiness, FCQ-T-r) between clusters. In addition, we conducted a Pearson’s Chi-squared test regarding group allocation in the RCT. Cluster memberships did not differ between the active and the control group, *X*^*2*^ (2, *N* = 114) = 4.14, *p* = .126. Moreover, since only a few men participated in the present study, we conducted Fisher’s exact test instead of Pearson’s Chi-squared test regarding gender and cluster memberships. The test revealed a significant difference (*p* = .019, Fisher’s exact test). In Cluster 1, there were 41 females (77.4%) and 12 males (22.6%), while in Cluster 2, there were 34 females (97.1%) and only one male (2.9%), whereas in Cluster 3, there were 20 females (76.9%) and six males (23.1%).

**Table 1:** Differences in trait characteristics between clusters

Outcome	*SS*_*Num*_	*SS*_*Den*_	*F*	*p*	η^2^_g_
**Compliance**	0.15	1.16	7.57	< .001 *	.12
**SSES**	4.11	62.77	3.63	.030 *	.06
**SEES happiness**	2.08	29.29	3.94	.022 *	.07
SEES anger	1.33	50.50	1.46	.236	.03
SEES anxiety	0.55	48.83	0.63	.535	.01
SEES sadness	1.61	60.57	1.47	.234	.03
DEBQ external	116.01	4104.06	1.57	.213	.03
DEBQ emotional	469.58	9104.93	2.81	.065	.05
DEBQ restrained	90.27	6021.36	0.83	.438	.02
**FCQ-T-r**	2017.62	16,619.16	6.74	.002 *	.11
BMI	20.34	1544.92	0.73	.484	.01

*N* = 114. The degrees of freedom numerator = 2 in all cases. The degrees of freedom denominator = 111 in all cases. *SS*_*Num*_ indicates sum of squares numerator. *SS*_*Den*_ indicates sum of squares denominator. η^2^_g_ indicates generalised eta-squared. *Compliance* completion rate of ecological momentary assessment questionnaires, *SSES* Salzburg Stress Eating Scale, *SEES* Salzburg Emotional Eating Scale, *DEBQ* Dutch Eating Behavior Scale, *FCQ-T-r* Food Cravings Questionnaire Trait reduced, *BMI* Body mass index. *P*-values with an asterisk indicate statistical significance at the *p* < .05 level. Outcomes written in bold indicate significant differences

## Discussion

The present study applied the novel analytical approach of latent class vector-autoregression (LCVAR) to affective eating behaviour research. We aimed to explore inter-individual patterns of lagged associations between momentary measures of affect, stress (coping), hunger, and food craving. We analysed 14 days of EMA data of 115 diet-interested individuals. The LCVAR resulted in three clusters with three lags each. Lastly, we characterised the clusters by means of trait-level data and completion rates of EMA questionnaires. Overall, the LCVAR results highlight the substantial variability in the relationships between affective states and hunger/food craving. Results support the assumption that temporal patterns of associations between affective and eating-related states might be similar across some individuals who could be clustered to form subgroups within a population. This appears plausible given the concept of ‘individual differences’ regarding the influence of affective states on eating behaviours [17, 18].

### Characterisation of clusters

In the following, we will characterise the three clusters using the LCVAR coefficients (Fig. 1), the daytime courses (Fig. 2) and the results of the ANOVAs and Tukey HSD, (see also Table 1 and Fig. 3). We will discuss the clusters in reverse order beginning with Cluster 3. Firstly, Cluster 3, the ‘smallest’ cluster containing 22.6% of participants, showed the most pronounced coefficients regarding associations between affective states and hunger/craving (see the first two rows in the heatmap in Fig. 1). Higher food cravings in Cluster 3 preceded feeling less calm (at one and two lags) and lower anticipated stress coping. By contrast, higher hunger was followed by feeling less depressed. Thus, in this cluster, hunger and craving appear to have specific and partially different associations with subsequent mood. Previous research has already differentiated effects of hunger and craving in that hunger might positively influence goal-congruent eating, while craving might have slightly negative influences on goal-congruent eating [14]. This is in line with the literature drawing further distinctions between the two [44]: e.g., hunger was negatively related to snack food consumption when taking craving intensity into account [45]. Based on the ANOVAs, Cluster 3 might be considered hosting *‘healthy eaters’*: Participants in Cluster 3 had the lowest mean FCQ-T-r score of all clusters (thus most likely included individuals with rather low trait food craving) and descriptively showed the lowest mean state cravings at all six intra-day measurement time points (Fig. 2). In addition, Fig. 3 illustrates that Cluster 3 showed balanced happiness eating (in this case: mostly eating as usual when feeling happy) and balanced stress eating (in this case: mostly eating slightly less to eating as usual when stressed). Importantly, contrary to what we had expected in general, we mainly observed associations between hunger/food craving and *subsequent* affective states (‘emotional about eating’), i.e., the opposite direction as traditionally assessed via the above-mentioned trait questionnaires (‘emotional eating’ [46], please also see the General discussion and methodological implications below). Lastly, from a methodological point of view, results might be influenced by a high compliance: The subsequent ANOVAs revealed that Cluster 3 had the significantly highest mean completion rate of EMA questionnaires of all three clusters.

Secondly, Cluster 2 (containing 31.3% of all participants) could be described as the ‘*affectively troubled’* cluster, based on rather pronounced associations between affect and eating behaviour, which might not be revealed at first glance by the coefficient heatmap (Fig. 1). However, Cluster 2 showed the highest mean negative affect scores and the lowest mean positive affect scores of all clusters in the ‘daytime courses’ (Fig. 2). Interestingly, results suggest a simultaneous increase in food craving and negative affect as well as a decrease in positive affect (which were most pronounced in Cluster 2).^1^ Furthermore, the ANOVAs and the post hoc contrasts indicate that Cluster 2 might be the subgroup with the lowest happy eating (SEES happiness) and the highest stress-eating (SSES) traits (see also Fig. 3) which might correspond to the above-mentioned daytime courses. Additionally, although not differing significantly from Cluster 1, Cluster 2 showed a relatively high mean value of trait craving (FCQ-T-r [47]), especially compared to Cluster 3. Interestingly, the ratio of males in Cluster 2 was notably smaller than in the other two clusters. Considering the above-mentioned results of happy eating and stress-eating in Cluster 2, results correspond to previous findings that women might show higher stress-eating than men (thus the high value in this cluster), while men might show higher happy eating than women (thus the low mean value in this cluster) [20, 21].

Lastly, Cluster 1 is characterised by small coefficients that do not suggest strong lagged associations between these two eating-related variables and affective states (see first two rows as well as the two columns before the lag lines). The results of the ANOVAs and the post hoc contrasts underline the assumption of Cluster 1 being the ‘*average’* subgroup within our sample, as the main scores of trait craving (FCQ-T-r), stress-eating (SSES), happy eating (SEES happiness) and the main completion rate of answered EMA questionnaires are mostly between those of Cluster 2 and 3.

### General discussion and methodological implications

Most notably, associations of eating behaviours (hunger and craving) with *subsequent* affective states were stronger than the reverse direction of association. Although most trait questionnaires and most research on emotional eating focus on emotion-induced eating behaviour [11, 48, 49], the reverse direction, i.e., affective changes in response to eating or food stimuli, is equally plausible and has also been documented [17, 50, 51]. The present results thus suggest that future research should routinely consider both directions of association between affect and appetitive behaviours. With the reverse direction, the question of actual food intake is pressing, which however was not included in the present study, as only momentary measures (instead of retrospective ones) were of interest. Yet, it is reasonable to assume that hunger and craving peak around lunch and dinner time and thus might peak in food intake which has been previously suggested by correlational data [44]. Interestingly, craving was negatively associated with subsequently feeling relaxed in Cluster 3, suggestive of potential non-rewarding food intake experiences, yet it was positively associated in Cluster 1 and 2. These between-cluster differences suggest that not all individuals respond to cravings (and potentially food intake) in a similar manner: For some individuals, (yielding to) food cravings might increase positive affect, for instance because of reward effects [52]. In this context, diet-interested individuals (as in the present study) might also crave *healthy* foods [45] that may be diet-goal-congruent. In contrast, other individuals might respond to cravings with increased negative affect or decreased positive affect, either because cravings might be perceived as potential threats to diet / weight goals [10] or because of actual subsequent consumption of high calory (i.e., often diet-incongruent) foods [45]. Thus, differences in individual coefficients can be based on different subjective experiences, making multiple ‘concurrent’ explanations possible. Besides these specific differences between clusters, craving also showed similarities across clusters: The daytime courses (Fig. 2) permit insight into diurnal trends of hunger and craving. All three clusters showed similar trends with higher values of hunger around midmorning and late afternoon as well as higher values of cravings in the afternoon. Such general trends (‘M-curve’) are in accordance with the results of previous studies concerning hunger and craving [44] as well as latent classes of eating patterns [53].

Importantly, we observed variability both between and within clusters: In Cluster 3, both the direction and the strength of coefficients can be observed to change most prominently across lags. For instance, the association between hunger and feeling depressed was notable for one and two lags but decreased with three lags. Additionally, the relationship between momentary craving and stress coping appeared to be slightly negative for one lag but positive for two lags. The variability within clusters underlines the relevance of a well-considered temporal resolution when studying relationships between affective states and eating behaviour: Assessment frequency and length of intervals between (thus the length of lags) might influence the direction and strength of results obtained later. Therefore, it appears reasonable to sample with a high frequency to best capture potential lagged associations and at the same time keep questionnaires short to reduce participant burden [54]. Lastly, a notable finding is that up to three lags of associations contributed to the best fit of the cluster solution (and even to the second-best fitting model). This indicates that the correlations between momentary data can ‘spread’ (systematically) over several hours – at least within certain subgroups or individuals. Such insights may indicate regularity and potentially even predictability in the oscillation of appetitive-affective experience in (different subgroups of) individuals which might relate to predictive modelling or machine learning approaches as often used in the context of just-in-time adaptive interventions [55].

### Limitations and future research

We utilised LCVAR to analyse time-series data aiming to identify potential subgroups. However, the existence of subgroups (i.e., clusters) within our data is an assumption of our model. Moreover, our description and characterisation of the clusters were mainly based on eating-related variables while also all associations among affective states contributed to cluster separation. For instance, lagged associations between different affective states might have been equally or even more influential for cluster membership allocation. Our clusters might thus reflect between-subject differences in affective dynamics just as well as they do reflect affective-appetitive dynamics. Therefore, we aimed to characterise the clusters using *eating*-related trait questionnaires in subsequent ANOVAs, suggesting that cluster separation might be related to differences in affective eating behaviours. Clearly, future studies should characterise clusters also with affective (e.g., emotion regulation, stress coping) trait questionnaires. Furthermore, we explicitly focussed on diet-interested individuals since hunger and food craving can play important roles in dieting. However, results of this mostly young and female sample (which is not representative for the general population) have a limited generalisability. Moreover, we did not directly analyse actual eating behaviour (e.g., retrospectively reported amounts of consumed foods), but related appetitive states (hunger and craving). Therefore, we did not take into account whether participants consumed foods after reporting high hunger or cravings and which eating behaviours actually took place between two measurements. Although this might provide important information, especially in hindsight regarding the surprisingly reverse direction of effects observed, we had decided to focus on momentary measures of states that were assessed simultaneously. From an analytical point of view, at the time of this publication, LCVAR does not yet support nested structures (e.g., measurements within a day). Thus, each day’s last measurement was used to predict the next day’s first value. Therefore, coefficients in the present study might underestimate associations, as the ‘predictive power’ of such overnight associations might be lower than intra-day ones. However, we refrained from interpolating values to add them ‘between two days’, because it does not appear reasonable to estimate hunger and craving (but also affect) values for night hours. Moreover, we set the threshold to at least 50 repeated measures per person to ensure good performance of the LCVAR [29], though a higher number of participants and even more measurement time points might be beneficial in future studies. Lastly, because of the exploratory nature of the present study, the results need to be replicated in order to draw concrete conceptual implications.

## Conclusions

LCVAR appears to be a promising approach to investigate between-subject differences as well as variability in affect and stress-related eating behaviours. LCVAR enables researchers to visually explore temporal patterns in the clusters obtained: Specific associations between hunger/craving on the one hand and specific affective states on the other hand can be compared across lags and clusters. Thus, LCVAR makes specific, data-driven exploration of temporally lagged relationships possible and visible, both within and between clusters. In the present study, associations between hunger/craving and affective states differed across variables as well as across lags within and between clusters. Temporal associations between hunger/craving and *subsequent* affective states were more pronounced than vice versa. The present study revealed empirically how highly variable affect-eating-relationships are. We exemplified the potential of LCVAR with a set of affective and eating-related variables, however, the present paper is not meant as a ‘how-to-guide’ and other (subjective or objective) variables may of course be of great interest for health or clinical research. Different analytical approaches might be combined to fully exploit the potential of such time-series data analyses, both in a data-driven as well as theory-based way. Future applications to other populations, e.g., individuals with eating disorders, might reveal clearer ‘patterns’ of relationships between affective states and eating behaviour [56] and may also help to modify existing theories and develop novel ones.

## Supplementary Information

**Additional file 1.** Coefficients tables (Cluster 1, Cluster 2, Cluster 3).

**Additional file 2.** List of additional variables.

**Additional file 3.** Simulated random data LCVAR.

**Additional file 4.** Information regarding the present empirical study.

## Data Availability

The datasets used and/or analysed during the current study are available from the corresponding author on reasonable request.

## Footnotes

1 This finding is not visible in the heatmaps, as contemporaneous associations are not included, however, based on the daytime courses corresponding patterns might be plausible.
