# Supplementary material for: Clustering individuals’ temporal patterns of affective states, hunger, and food craving by latent class vector-autoregression
Source: Int J Behav Nutr Phys Act. 2022 May 21;19:57. doi: 10.1186/s12966-022-01293-1 (PMC9123755; doi:10.1186/s12966-022-01293-1)
Supplement: Supplementary file 1 — Additional file 1. Coefficients tables (Cluster 1, Cluster 2, Cluster 3). [file 12966_2022_1293_MOESM1_ESM.pdf]

	cheerful_1	enthusiastic_1	relaxed_1	calm_1	irritated_1	worried_1	depressed_1	bored_1	nervous / stressed_1	active_1	present coping_1	anticipated coping_1	hunger_1	craving_1
cheerful	0.245	-0.010	0.048	-0.009	0.021	0.026	0.007	0.038	-0.038	-0.038	-0.019	0.023	0.034	-0.029
enthusiastic	0.039	0.210	0.068	-0.001	0.015	0.039	-0.005	-0.001	-0.005	-0.020	0.034	-0.052	0.029	-0.030
relaxed	0.032	-0.012	0.157	0.064	0.047	0.027	-0.013	0.072	-0.054	-0.057	-0.042	0.044	0.008	0.004
calm	-0.001	0.001	0.044	0.200	0.039	-0.002	0.014	0.036	0.006	-0.070	-0.033	0.036	0.006	0.009
irritated	-0.015	0.004	0.026	0.016	0.261	-0.017	0.036	0.029	0.008	-0.002	-0.058	0.047	-0.021	-0.001
worried	0.011	-0.008	0.029	-0.032	-0.008	0.186	0.074	-0.033	0.018	0.011	0.068	-0.033	-0.011	-0.006
depressed	0.024	-0.003	0.015	-0.008	-0.009	0.069	0.250	-0.054	0.002	0.018	0.006	-0.026	-0.011	-0.009
bored	0.017	-0.002	0.020	-0.029	-0.019	-0.004	-0.037	0.182	0.047	0.019	0.014	-0.029	-0.024	0.019
nervous / stressed	0.008	0.010	-0.023	0.027	-0.012	0.007	-0.010	0.009	0.167	0.008	-0.045	0.036	-0.004	0.003
active	0.008	-0.002	0.028	-0.019	-0.007	0.009	0.061	-0.101	0.024	0.267	-0.044	0.027	-0.019	0.003
present coping	-0.011	-0.006	0.007	0.005	0.009	-0.024	0.029	0.080	-0.034	-0.034	0.247	0.050	0.018	0.000
anticipated coping	0.001	-0.011	-0.008	0.010	0.021	-0.020	0.014	0.060	-0.024	-0.030	0.038	0.262	0.019	0.000
hunger	-0.041	0.068	-0.022	-0.006	0.004	0.011	0.004	-0.054	0.017	-0.006	-0.064	0.001	0.047	0.021
craving	0.002	0.045	-0.029	-0.014	-0.004	0.011	-0.044	0.035	0.034	0.026	-0.057	0.031	-0.038	0.125

	cheerful_2	enthusiastic_2	relaxed_2	calm_2	irritated_2	worried_2	depressed_2	bored_2	nervous / stressed_2	active_2	present coping_2	anticipated coping_2	hunger_2	craving_2
cheerful	0.146	0.058	0.008	0.024	0.008	0.066	-0.023	0.062	0.018	-0.007	-0.075	0.100	0.014	-0.013
enthusiastic	-0.018	0.235	-0.003	0.047	0.000	0.033	0.021	0.060	0.016	-0.012	-0.045	0.068	0.007	-0.012
relaxed	-0.052	0.096	0.074	0.115	0.016	0.066	0.036	0.049	0.023	-0.023	-0.055	0.060	-0.011	-0.001
calm	-0.067	0.101	-0.010	0.201	0.002	0.041	0.016	0.031	-0.004	-0.039	-0.086	0.072	0.004	-0.008
irritated	-0.048	0.061	0.088	-0.022	0.142	0.020	0.007	0.052	-0.012	0.013	-0.026	0.033	-0.029	0.008
worried	0.023	-0.013	0.033	-0.019	0.021	0.120	0.057	-0.034	0.013	0.009	-0.031	0.037	0.010	-0.005
depressed	0.013	0.010	0.007	-0.015	-0.006	0.060	0.128	-0.039	0.018	0.009	0.051	-0.070	0.011	-0.006
bored	0.030	-0.001	0.006	-0.026	0.000	-0.012	-0.029	0.073	0.039	0.017	-0.002	-0.019	0.004	0.012
nervous / stressed	0.020	-0.004	0.022	-0.045	0.014	0.020	-0.018	-0.008	0.121	-0.014	-0.005	-0.043	0.007	0.000
active	0.028	-0.025	0.010	0.014	-0.006	0.029	-0.003	-0.015	-0.008	0.174	0.021	-0.046	0.013	0.005
present coping	-0.036	0.014	-0.005	0.018	0.002	0.033	0.009	0.036	-0.030	0.009	0.174	0.062	-0.016	0.013
anticipated coping	-0.028	0.012	0.006	0.021	0.002	0.035	-0.007	0.054	-0.009	0.014	-0.021	0.265	-0.026	0.012
hunger	0.026	-0.020	-0.034	0.033	-0.009	-0.004	0.030	-0.041	0.009	0.000	-0.009	0.025	0.080	0.037
craving	-0.046	0.006	0.003	0.002	0.017	-0.010	0.048	-0.060	-0.004	0.000	0.048	-0.015	0.024	0.086

	cheerful_3	enthusiastic_3	relaxed_3	calm_3	irritated_3	worried_3	depressed_3	bored_3	nervous / stressed_3	active_3	present coping_3	anticipated coping_3	hunger_3	craving_3
cheerful	0.167	0.054	0.030	-0.049	-0.018	-0.006	-0.021	0.118	0.020	0.047	0.011	0.033	-0.002	-0.010
enthusiastic	0.015	0.232	0.066	-0.047	-0.037	-0.036	-0.064	0.118	0.041	0.060	-0.013	-0.001	-0.005	-0.010
relaxed	-0.026	0.066	0.129	0.000	0.013	-0.006	0.013	0.052	-0.006	0.031	0.028	0.011	0.006	-0.010
calm	-0.040	0.056	0.039	0.112	-0.001	-0.020	0.023	0.056	-0.005	0.051	-0.011	0.096	-0.001	-0.003
irritated	0.086	-0.057	0.063	-0.057	0.117	0.019	-0.010	0.004	0.021	0.030	-0.046	0.064	-0.027	0.034
worried	0.032	-0.027	-0.002	-0.013	-0.001	0.092	0.050	-0.048	0.015	0.007	-0.007	0.005	0.004	0.011
depressed	-0.003	-0.033	-0.012	0.051	0.011	0.054	0.142	-0.099	0.017	-0.007	0.000	-0.014	0.009	0.002
bored	0.000	0.000	-0.004	0.022	0.013	0.034	0.030	0.051	0.035	-0.032	0.000	-0.001	-0.003	-0.009
nervous / stressed	0.010	-0.008	0.000	0.009	-0.007	-0.022	0.032	0.047	0.038	-0.011	0.005	0.010	0.016	-0.011
active	0.022	-0.038	-0.031	0.041	-0.007	0.007	0.014	-0.082	0.020	0.121	-0.027	-0.012	-0.008	0.034
present coping	0.006	0.006	0.038	-0.057	-0.014	0.007	0.002	0.098	-0.035	0.009	0.210	0.038	0.010	-0.017
anticipated coping	0.016	-0.005	0.036	-0.053	-0.009	0.007	0.007	0.095	-0.050	0.028	0.032	0.206	0.028	-0.022
hunger	0.059	-0.032	0.001	-0.009	-0.029	0.017	-0.021	0.000	0.017	0.054	-0.061	0.087	0.101	0.011
craving	0.023	-0.017	0.039	0.004	0.004	-0.036	-0.033	0.061	-0.030	0.055	-0.060	0.049	0.029	0.087

Cluster 1 coefficients for one (_1), two (_2), and three (_3) lags.

	cheerful_1	enthusiastic_1	relaxed_1	calm_1	irritated_1	worried_1	depressed_1	bored_1	nervous / stressed_1	active_1	present coping_1	anticipated coping_1	hunger_1	craving_1
cheerful	0.254	0.030	-0.053	0.044	0.017	0.019	0.030	-0.061	0.049	0.002	-0.033	0.048	0.018	-0.006
enthusiastic	-0.007	0.220	-0.018	0.038	0.034	-0.003	0.024	-0.035	0.034	0.018	-0.020	0.047	0.029	0.017
relaxed	0.075	0.016	0.168	0.003	0.011	-0.026	0.032	-0.011	0.022	-0.013	0.064	-0.028	0.001	0.027
calm	0.045	0.029	0.028	0.183	0.026	-0.031	0.039	-0.021	0.015	-0.004	0.049	-0.054	0.024	0.004
irritated	-0.017	0.088	-0.032	0.002	0.171	0.076	0.008	-0.075	0.030	0.037	0.027	0.044	0.004	-0.040
worried	0.013	-0.036	0.019	0.010	0.005	0.219	0.003	0.013	-0.006	0.008	-0.040	0.041	0.033	-0.021
depressed	0.029	0.000	-0.027	0.014	-0.023	0.029	0.238	0.007	-0.010	0.017	-0.060	0.026	0.002	-0.002
bored	-0.058	0.030	0.027	-0.033	0.002	-0.018	-0.013	0.313	-0.010	-0.024	-0.056	0.021	-0.012	0.000
nervous / stressed	-0.011	0.007	0.052	-0.002	0.012	0.009	-0.037	0.035	0.174	-0.020	-0.062	0.022	0.019	0.005
active	0.005	0.014	0.002	-0.045	0.013	0.049	0.035	0.000	0.032	0.249	0.018	0.030	0.002	-0.020
present coping	0.019	-0.010	-0.027	0.057	0.017	0.042	0.006	-0.050	0.016	0.017	0.213	0.022	0.008	-0.009
anticipated coping	-0.005	0.014	-0.021	0.012	0.009	0.034	0.009	-0.056	0.033	0.019	-0.019	0.292	-0.004	0.006
hunger	-0.001	0.002	-0.043	-0.021	0.055	-0.013	-0.019	0.029	-0.004	0.053	-0.006	0.055	0.045	0.034
craving	-0.032	0.054	-0.034	-0.004	0.011	-0.021	0.034	0.018	0.025	0.011	0.071	-0.032	0.019	0.153

	cheerful_2	enthusiastic_2	relaxed_2	calm_2	irritated_2	worried_2	depressed_2	bored_2	nervous / stressed_2	active_2	present coping_2	anticipated coping_2	hunger_2	craving_2
cheerful	0.162	-0.023	0.065	-0.014	0.015	-0.020	0.037	0.020	0.026	-0.044	0.108	-0.082	0.001	0.015
enthusiastic	0.024	0.096	0.068	-0.022	0.013	0.017	0.016	0.018	0.040	-0.046	0.008	0.005	0.007	0.010
relaxed	0.020	-0.025	0.158	0.016	0.041	0.011	-0.018	0.018	0.044	-0.038	-0.018	0.044	-0.012	-0.003
calm	0.000	-0.023	-0.002	0.202	0.022	0.001	-0.036	0.049	0.032	-0.014	0.017	0.029	0.015	-0.001
irritated	0.050	-0.008	0.025	0.001	0.096	-0.006	0.033	0.024	0.028	-0.056	0.048	-0.073	0.014	-0.001
worried	0.023	0.032	-0.028	0.009	0.021	0.171	0.028	-0.033	0.013	-0.006	0.006	-0.036	0.005	0.013
depressed	-0.004	0.023	-0.045	0.014	0.018	0.038	0.184	-0.024	-0.024	0.023	0.017	0.008	-0.017	0.035
bored	0.037	0.004	-0.034	0.038	0.014	0.043	-0.013	0.161	0.006	0.025	0.002	-0.057	-0.023	0.016
nervous / stressed	0.024	0.006	-0.019	0.069	-0.022	0.052	-0.003	0.019	0.154	0.022	0.059	-0.054	0.011	0.003
active	0.045	0.001	-0.065	-0.010	-0.013	-0.023	0.075	-0.016	0.006	0.147	0.049	-0.001	0.013	0.054
present coping	0.001	-0.032	0.016	0.001	-0.025	-0.029	0.002	0.010	0.021	0.021	0.221	0.045	0.009	0.013
anticipated coping	0.007	-0.038	0.014	0.018	-0.029	-0.021	0.010	-0.004	0.020	0.016	0.036	0.226	0.005	0.007
hunger	-0.030	0.028	-0.013	0.057	0.019	0.044	0.036	-0.070	-0.036	-0.026	0.041	-0.080	0.091	0.040
craving	-0.003	0.006	0.035	-0.017	-0.008	0.010	0.043	-0.007	0.004	-0.032	0.048	-0.050	0.011	0.150

	cheerful_3	enthusiastic_3	relaxed_3	calm_3	irritated_3	worried_3	depressed_3	bored_3	nervous / stressed_3	active_3	present coping_3	anticipated coping_3	hunger_3	craving_3
cheerful	0.163	0.020	0.033	-0.010	-0.039	0.023	0.024	0.006	0.023	-0.014	0.023	-0.029	-0.010	0.019
enthusiastic	0.020	0.171	0.030	-0.003	-0.026	0.012	0.027	0.012	0.014	-0.016	-0.010	0.007	-0.003	0.032
relaxed	0.032	0.014	0.119	0.016	-0.012	0.000	0.026	0.049	0.043	-0.035	-0.033	0.041	-0.008	0.019
calm	-0.001	0.023	-0.001	0.128	-0.042	0.009	0.064	0.010	0.065	-0.043	0.047	-0.018	-0.012	0.006
irritated	0.053	0.003	0.027	-0.039	0.031	0.050	-0.009	0.022	-0.008	0.014	0.105	-0.095	0.002	0.027
worried	0.005	0.005	0.010	0.014	0.003	0.118	-0.020	-0.005	0.003	0.044	-0.030	0.011	0.017	0.006
depressed	0.025	-0.014	0.040	0.001	-0.023	0.042	0.097	0.031	-0.018	0.036	0.007	-0.018	-0.009	-0.003
bored	0.024	0.016	-0.020	0.032	-0.016	-0.017	-0.013	0.156	0.016	0.003	-0.047	0.022	-0.003	0.030
nervous / stressed	-0.004	0.015	-0.025	0.031	0.027	0.009	0.016	-0.006	0.153	0.026	-0.038	0.060	-0.043	0.016
active	-0.026	-0.038	0.025	0.017	-0.011	0.005	-0.073	0.018	-0.035	0.205	0.035	0.015	-0.027	0.015
present coping	-0.002	0.000	0.006	-0.020	-0.023	-0.023	0.039	-0.013	0.008	0.027	0.207	0.009	-0.001	-0.012
anticipated coping	-0.016	0.022	0.018	-0.029	-0.030	-0.014	0.020	0.016	0.003	0.028	0.058	0.171	-0.015	-0.013
hunger	0.029	0.031	0.008	-0.014	0.001	0.053	-0.035	0.012	0.019	0.072	0.092	-0.100	0.094	-0.005
craving	0.016	0.092	0.024	-0.006	-0.045	0.032	-0.002	0.027	-0.033	0.084	0.060	-0.043	-0.009	0.128

Cluster 2 coefficients for one (_1), two (_2), and three (_3) lags.

	cheerful_1	enthusiastic_1	relaxed_1	calm_1	irritated_1	worried_1	depressed_1	bored_1	nervous / stressed_1	active_1	present coping_1	anticipated coping_1	hunger_1	craving_1
cheerful	0.320	-0.012	0.072	-0.090	0.055	0.002	0.073	0.079	0.021	-0.115	0.098	-0.052	0.006	-0.018
enthusiastic	0.022	0.346	-0.017	0.006	0.011	0.016	0.038	0.110	-0.004	-0.041	0.078	-0.055	0.005	-0.022
relaxed	0.024	-0.068	0.302	0.056	0.035	0.048	0.026	0.102	-0.076	-0.100	-0.003	-0.043	0.001	-0.007
calm	0.008	-0.048	0.140	0.258	0.035	0.081	-0.018	0.123	-0.081	-0.050	-0.056	0.009	-0.011	-0.002
irritated	0.025	0.065	-0.055	0.013	0.247	0.044	0.077	0.120	0.077	-0.113	0.041	-0.056	-0.014	-0.056
worried	0.009	0.010	-0.017	0.009	0.021	0.148	-0.010	-0.012	0.049	0.062	-0.057	0.048	-0.008	0.029
depressed	0.003	-0.006	0.005	0.013	-0.013	0.095	0.149	-0.073	0.013	0.060	0.034	-0.097	-0.031	-0.001
bored	-0.001	-0.015	-0.012	0.028	0.006	0.070	-0.050	0.140	-0.018	0.058	-0.037	0.025	0.000	0.001
nervous / stressed	-0.012	-0.003	-0.018	0.014	-0.004	0.032	-0.011	-0.028	0.262	0.029	-0.053	0.072	0.018	0.010
active	-0.019	0.024	-0.049	0.023	-0.001	-0.022	-0.036	-0.038	0.052	0.332	0.006	-0.037	-0.027	0.016
present coping	-0.029	-0.018	0.053	-0.038	0.025	-0.034	0.011	0.028	-0.001	-0.041	0.274	0.032	-0.007	0.014
anticipated coping	-0.028	-0.023	0.011	0.002	0.012	-0.058	0.006	0.032	-0.006	-0.012	0.057	0.263	-0.014	0.017
hunger	-0.005	0.041	-0.042	-0.005	-0.020	0.019	-0.140	-0.007	0.085	0.053	-0.117	0.091	0.059	-0.005
craving	0.049	0.028	0.145	-0.154	-0.009	-0.085	-0.102	0.066	0.055	0.100	-0.072	-0.020	0.037	0.090

	cheerful_2	enthusiastic_2	relaxed_2	calm_2	irritated_2	worried_2	depressed_2	bored_2	nervous / stressed_2	active_2	present coping_2	anticipated coping_2	hunger_2	craving_2
cheerful	0.238	-0.017	-0.010	0.035	-0.025	0.084	-0.027	-0.014	-0.016	0.060	-0.020	-0.050	-0.002	-0.020
enthusiastic	-0.008	0.201	-0.011	0.044	-0.028	0.106	-0.011	-0.033	0.048	0.027	-0.031	0.008	0.017	-0.013
relaxed	-0.045	0.053	0.080	0.143	0.007	0.074	-0.001	0.017	0.055	0.048	0.066	-0.089	-0.008	0.018
calm	-0.016	0.041	-0.058	0.267	-0.003	0.087	-0.037	0.001	0.096	0.072	-0.014	0.027	-0.002	0.011
irritated	0.059	-0.068	0.085	-0.034	0.136	-0.003	-0.039	-0.036	-0.066	0.085	-0.021	-0.068	-0.037	-0.018
worried	0.028	0.015	-0.043	0.037	-0.019	0.011	0.029	0.027	0.061	0.021	-0.013	0.026	0.006	-0.009
depressed	-0.003	-0.011	-0.015	-0.034	0.025	-0.043	0.107	-0.058	0.003	0.018	0.012	-0.012	-0.025	-0.001
bored	0.011	0.009	-0.009	0.007	0.015	0.019	0.051	0.072	0.007	0.002	-0.060	0.048	-0.002	0.004
nervous / stressed	0.007	0.027	-0.004	0.030	-0.007	-0.038	0.073	-0.037	0.181	0.019	0.066	-0.032	0.029	-0.002
active	0.005	-0.040	0.021	0.008	0.017	-0.041	-0.004	-0.091	0.035	0.191	0.036	-0.055	-0.016	0.000
present coping	-0.027	0.033	0.013	-0.001	-0.019	0.018	-0.032	0.053	0.010	0.014	0.180	0.031	-0.003	0.009
anticipated coping	-0.026	0.026	-0.011	0.021	-0.023	0.058	-0.020	0.056	-0.009	-0.007	0.021	0.201	-0.012	-0.001
hunger	0.066	0.028	-0.007	-0.048	-0.048	0.022	-0.149	-0.062	0.086	0.064	0.049	-0.053	0.101	-0.036
craving	-0.007	0.025	0.143	-0.156	-0.018	0.005	-0.057	0.019	0.035	0.049	0.172	-0.157	-0.012	0.097

	cheerful_3	enthusiastic_3	relaxed_3	calm_3	irritated_3	worried_3	depressed_3	bored_3	nervous / stressed_3	active_3	present coping_3	anticipated coping_3	hunger_3	craving_3
cheerful	0.237	0.006	-0.030	0.046	-0.004	0.041	-0.004	0.039	0.020	-0.010	-0.013	-0.079	-0.001	0.011
enthusiastic	0.016	0.220	-0.026	0.020	-0.013	0.092	0.054	0.040	-0.015	-0.103	0.110	-0.155	-0.023	0.002
relaxed	0.012	0.011	0.142	0.060	0.011	-0.054	0.020	0.045	-0.059	0.041	0.051	0.020	-0.027	0.050
calm	-0.017	0.033	0.033	0.173	0.025	-0.023	-0.009	0.013	-0.031	0.020	-0.045	0.093	-0.040	0.032
irritated	0.043	-0.035	-0.066	0.083	0.157	0.059	0.079	-0.017	0.056	-0.077	-0.007	-0.070	-0.014	-0.017
worried	-0.031	0.018	-0.018	-0.003	0.024	0.067	0.019	0.085	0.027	-0.022	-0.017	0.045	0.029	-0.018
depressed	0.011	0.043	0.003	-0.002	-0.012	0.009	0.190	0.006	0.022	-0.014	-0.065	0.023	-0.007	0.000
bored	-0.024	0.027	0.007	-0.008	0.002	-0.011	0.024	0.099	0.010	-0.041	0.072	-0.065	0.005	-0.007
nervous / stressed	0.029	0.017	-0.023	-0.002	0.001	-0.026	0.010	0.026	0.130	0.014	0.018	-0.002	-0.003	0.015
active	0.016	0.006	-0.030	0.010	-0.028	-0.004	0.043	-0.032	0.023	0.140	-0.059	0.006	0.021	-0.017
present coping	-0.003	-0.011	-0.017	-0.007	-0.021	0.019	-0.014	-0.005	0.026	-0.017	0.147	0.069	-0.010	0.006
anticipated coping	-0.006	-0.003	-0.072	0.034	-0.022	0.028	-0.021	-0.015	0.030	-0.026	-0.005	0.230	-0.009	-0.007
hunger	0.010	0.034	-0.021	-0.039	-0.055	0.067	-0.027	-0.058	0.052	-0.031	-0.047	-0.078	0.050	0.014
craving	-0.040	0.024	0.068	-0.054	-0.039	0.011	0.012	-0.009	-0.007	0.010	0.139	-0.113	-0.006	0.127

Cluster 3 coefficients for one (_1), two (_2), and three (_3) lags.
