# Supplementary material for: Clustering individuals’ temporal patterns of affective states, hunger, and food craving by latent class vector-autoregression
Source: Int J Behav Nutr Phys Act. 2022 May 21;19:57. doi: 10.1186/s12966-022-01293-1 (PMC9123755; doi:10.1186/s12966-022-01293-1)
Supplement: Supplementary file 2 — Additional file 2. List of additional variables. [file 12966_2022_1293_MOESM2_ESM.pdf]

Clustering individuals' temporal patterns of affective states, hunger, and food craving by latent class vector-autoregression

Pannicke, Blechert, Reichenberger & Kaiser (2022)

Supplementary materials: List of additional variables

Additional variables measured but not used in the present study

- Subjective sleep quality [only in the morning]
- Daily self-efficacy regarding eating behaviour
- Extent and point in time of retrospective food cravings since the last beep
- Retrospective food reports (amount, satisfaction with amount, food categories, type of meal, location of meal, eating alone or in company, time of meal)
- Retrospective estimate of impulsivity regarding eating behaviour
- Retrospective report of stressful event (extent and importance)
- Retrospective estimate of eating behaviour on the respective day (goal-congruent behaviour, planning, motivation, satisfaction) [only in the evening]
- Prospective estimate of eating behaviour for the next day (goal-congruent behaviour) [only in the evening]
- Retrospective report of food shopping [only in the evening]
- Report regarding eating behaviour related short messages: Brief evaluation [only in the evening, only active group]
- Retrospective daily self-efficacy regarding eating behaviour [only in the evening]
