# Supplementary material for: Clustering individuals’ temporal patterns of affective states, hunger, and food craving by latent class vector-autoregression
Source: Int J Behav Nutr Phys Act. 2022 May 21;19:57. doi: 10.1186/s12966-022-01293-1 (PMC9123755; doi:10.1186/s12966-022-01293-1)

Clustering individuals' temporal patterns of affective states, hunger, and food craving by latent class vector-autoregression

Pannicke, Blechert, Reichenberger & Kaiser (2022)

Supplementary materials: Simulated random data LCVAR

Randomly simulated data (suggested by a reviewer of this paper)

- 14 variables (scale: 0 – 100)
- 115 'individuals'
- 84 observations of all variables per 'individual'
- All other settings were the same as in our original analysis.

Results

Best model fit: 2 clusters with one lag each

Cluster 1: 47%, Cluster 2: 53% of individuals

Notably smaller coefficients and no clear pattern of associations

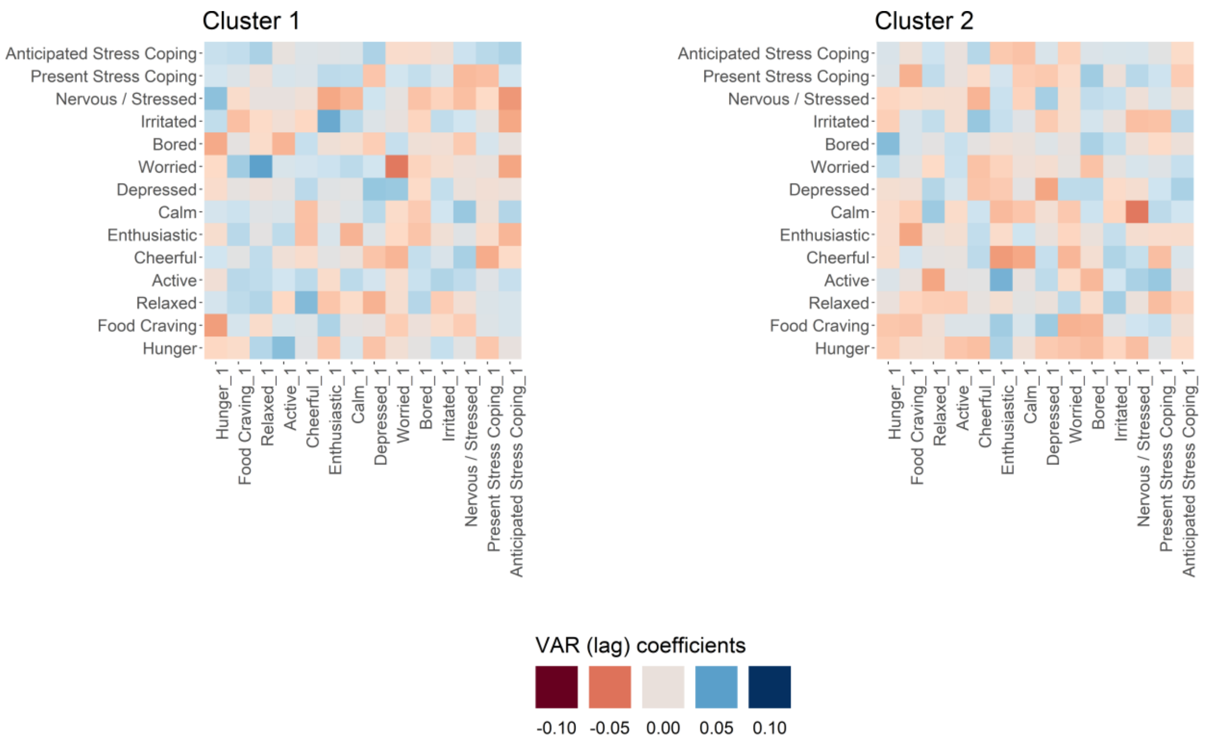

Supplement: Supplementary file 3 — Additional file 3. Simulated random data LCVAR. [file 12966_2022_1293_MOESM3_ESM.pdf]
