# Supplementary material for: Clustering individuals’ temporal patterns of affective states, hunger, and food craving by latent class vector-autoregression
Source: Int J Behav Nutr Phys Act. 2022 May 21;19:57. doi: 10.1186/s12966-022-01293-1 (PMC9123755; doi:10.1186/s12966-022-01293-1)
Supplement: Supplementary file 4 — Additional file 4. Information regarding the present empirical study. [file 12966_2022_1293_MOESM4_ESM.pdf]

Clustering individuals' temporal patterns of affective states, hunger, and food craving by latent class vector-autoregression

Pannicke, Blechert, Reichenberger & Kaiser (2022)

Information regarding the present empirical study

We recruited diet-interested individuals by means of a study announcement via e-mail and by word of mouth at several universities across Austria and Germany as well as via Facebook and Twitter. Individuals intending to 'maintain or reduce their body weight' were invited to participate in the study, since hunger and food craving can play important roles in these populations. This information can be found in the 'Procedure' part of the 'Methods' section in our manuscript.

Our sample consisted of mostly young and female adults who were diet-interested. Therefore, the sample has a limited generalisability with regard to all dieting adults. In the 'Limitations and future research' part of the 'Discussion' section of our manuscript, we acknowledged this information.

We included only participants who answered at least 60% of all ecological momentary assessment questionnaires (i.e., at least 50 observations per individual) in the analyses. We set this threshold and imputed missing data to ensure good performance of the clustering method (latent class vector-autoregression).
